# Unlicensed medical practitioners in tribal dominated rural areas of central India: bottleneck in malaria elimination

**DOI:** 10.1186/s12936-020-3109-z

**Published:** 2020-01-14

**Authors:** Mrigendra Pal Singh, Sunil Kumar Chand, Kalyan Brata Saha, Neetiraj Singh, Ramesh C. Dhiman, Lora L. Sabin

**Affiliations:** 1 ICMR-National Institute of Malaria Research Field Unit Jabalpur, NIRTH Campus, Nagpur Road, Post Garha, Jabalpur, 482003 Madhya Pradesh India; 2 ICMR-National Institute of Research in Tribal Health, NIRTH Campus, Nagpur Road, Post Garha, Jabalpur, 482003 Madhya Pradesh India; 3 Tribal Development and Research Institute, Ministry of Tribal Affairs, Government of Madhya Pradesh, 35 Shyamla Hills, Bhopal, India; 4 ICMR-National Institute of Malaria Research, Sector 8, Dwarka, New Delhi 110077 India; 5 Department of Global Health, Boston University School of Public Health, 801 Massachusetts Ave, Boston, MA USA

**Keywords:** Irrational use of antimalarial drugs, Monotherapy of artemisinin, *Plasmodium falciparum*, Tribal malaria, Unlicensed medical practitioner

## Abstract

**Background:**

In India, Accredited Social Health Activists (ASHAs) deliver services for diagnosis and treatment of malaria, although unlicensed medical practitioners (UMPs) (informal health providers) are most preferred in communities. A cross sectional survey was conducted to: (i) assess knowledge and treatment-seeking practices in the community, and (ii) explore the diagnosis and treatment practices related to malaria of UMPs working in rural and tribal-dominated high malaria endemic areas of central India, and whether they adhere to the national guidelines.

**Methods:**

A multi-stage sampling method and survey technique was adopted. Heads of the households and UMPs were interviewed using a structured interview schedule to assess knowledge and malaria treatment practices.

**Results:**

Knowledge regarding malaria symptoms was generally accurate, but misconceptions emerged related to malaria transmission and mosquito breeding places. Modern preventive measures were poorly accessed by the households. UMPs were the most preferred health providers (49%) and the first choice in households for seeking treatment. UMPs typically lacked knowledge of the names of malaria parasite species and species-specific diagnosis and treatment. Further, irrational use of anti-malarial drugs was common.

**Conclusions:**

UMPs were the most preferred type of health care providers in rural communities where health infrastructure is poor. The study suggests enhancing training of UMPs on national guidelines for malaria diagnosis and treatment to strengthen their ability to contribute to achievement of India’s malaria elimination goals.

## Background

The World Health Organization (WHO) recommends use of artemisinin-based combination therapy (ACT) for treatment of uncomplicated *Plasmodium falciparum* malaria [1]. In India, ACT has been the first-line treatment of confirmed *P. falciparum* malaria nationwide since 2010, after artemisinin monotherapy was banned in 2009 [2]. Chloroquine is the first-line drug for treatment of confirmed *Plasmodium vivax* malaria [3]. Primaquine is also recommended in a single dose for *P. falciparum* and a 14-day dose for *P. vivax* [3]. Malaria is the most common cause of fever in tribal-dominated areas of central India [4]; *P. falciparum* and *P. vivax* are the most common species in this area, with *P. falciparum* the most dominant species [4]. *Anopheles culicifacies* is the primary vector of malaria in central India [5].

People living in tribal-dominated hilly forested areas are highly vulnerable to malarial infections due to geo-climatic factors and poor access to health facilities. Further, in many communities, a poor understanding of the aetiology of malaria and various cultural practices add to this vulnerability [6, 7]. Low literacy levels and poor economic conditions also pose constraints for prompt diagnosis and treatment-seeking in the community [8]. Under the umbrella of the National Rural Health Mission, a cadre of female community volunteers known as Accredited Social Health Activists (ASHAs) was created to deliver rural health care services, mainly related to maternal and child health and vector borne diseases [2]. However, in many rural communities, particularly in tribal areas, unlicensed medical practitioners (UMPs) (informal health providers) are most preferred providers, including for treatment of malaria [7].

According to an estimate in India, among private health providers 57% had no recognized medical qualification, but they practice some form of allopathic medicine [9, 10]. They mainly treat common illnesses like diarrhoea, fever, malaria, vomiting, rashes, joint pains, respiratory distress, abdominal pain, flu, and typhoid. Poor health infrastructure and absenteeism among formal health workers make rural UMPs easier to reach [11], which has resulted in a treatment system in rural tribal areas whereby UMPs play a significant role as health service providers. Although, they do not have the legal right or status to provide health care services, their role is critical because they work in remote rural areas where medical facilities are scarce [11].

A cross sectional survey was conducted with two main objectives: (i) to assess the knowledge and treatment-seeking practices of the community, and (ii) to explore the diagnosis and treatment practices related to malaria of UMPs working in rural and tribal-dominated high malaria endemic areas of central India, and its adherence to the national guidelines.

## Methods

### Sample size and sampling methods

In India, about 50% of the population receive health care services for febrile illness from informal providers [7, 11, 12]. Based on this proportion, a sample size was determined for household-level interviews to assess the utilization of health care services. Considering 5% precision and a design effect of 2, with an additional 30% to account for non-responses, a sample of 1000 households was determined for the household survey.

**Fig. 1:**
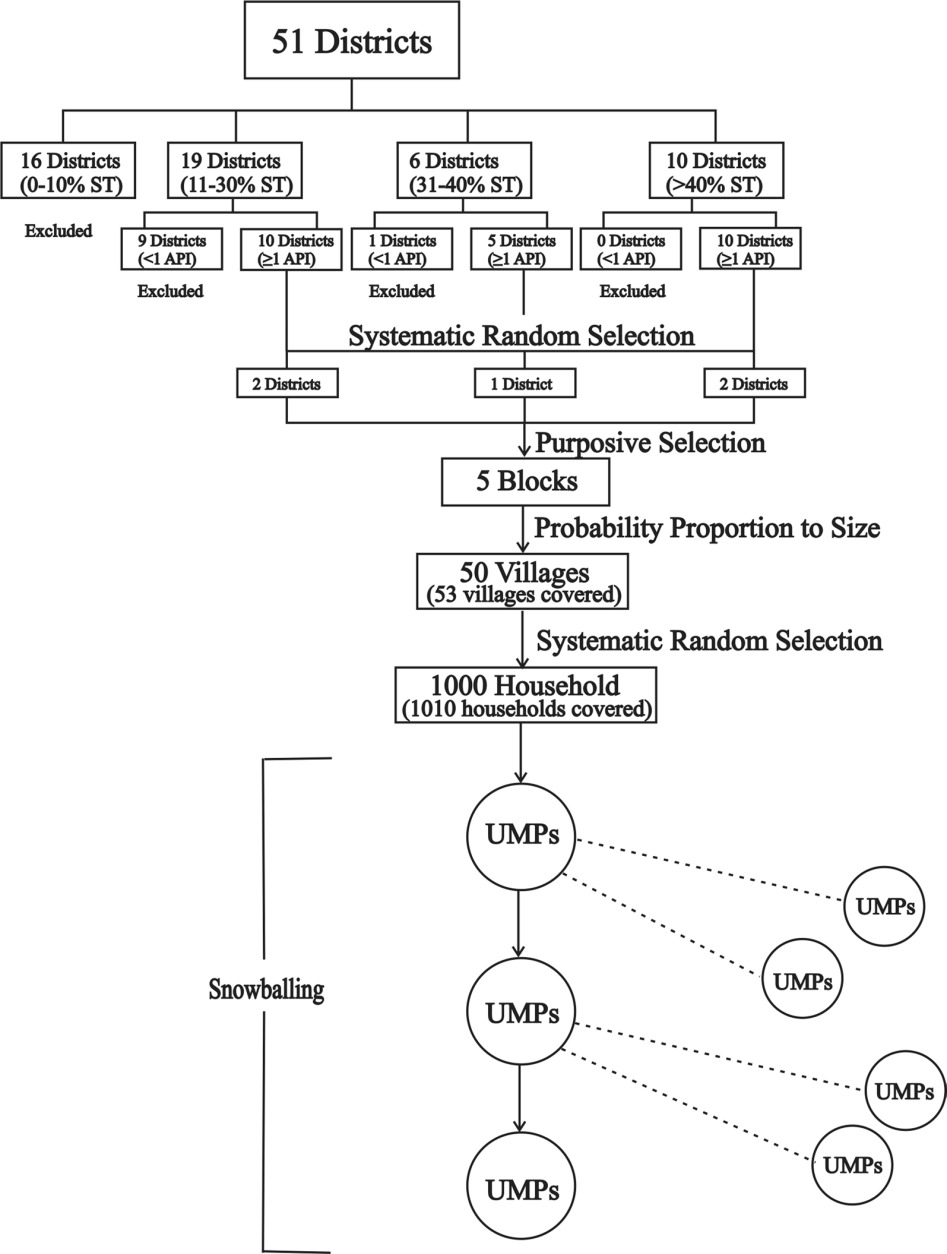
Sampling method for selection of study participants. ST: Scheduled tribe; API: Annual parasite incidence; UMPs: Unlicensed medical practitioners

**Fig. 2:**
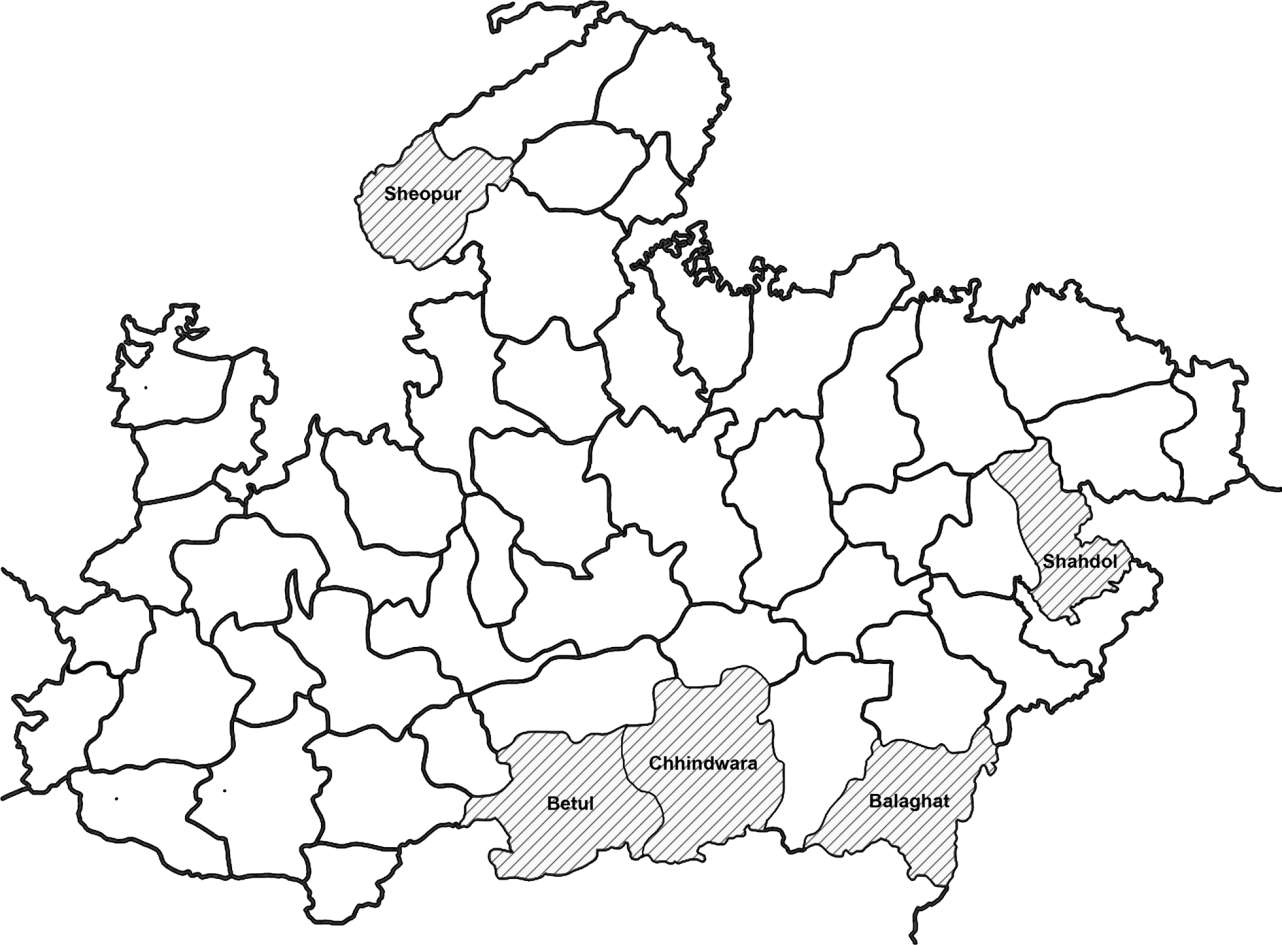
Map of Madhya Pradesh (central India) showing study districts (Source: National Informatics Centre, Madhya Pradesh State. Available at http://mp.nic.in/district.asp)

A multi-stage sampling method was adopted (Fig. 1). First, all of the 51 districts of Madhya Pradesh were grouped into four clusters based on proportion of tribal population, as follows: 0–10% (16 districts), 11–30% (19 districts), 31–40% (6 districts) and over 40% (10 districts). Cluster one districts were excluded due to their low proportions of tribal residents. Districts in each cluster which had less than one annual parasite incidence (API), defined as the number of malaria-positive cases per one thousand populations during one calendar year, were also excluded. Of the remaining districts, two from cluster two (Balaghat and Sheopur), one from cluster three (Chhindwara), and two from cluster four (Betul and Shahdol) were randomly selected for the household survey (Fig. 2). These five districts accounted for 10% of the total state population and 16% of the state’s tribal population [13]; they also contributed about 20% of malaria cases [14]. Second, one administrative block in each district was selected purposively based on the highest proportion of tribal population and malaria cases. Ten villages were then selected randomly from each block employing probability proportional to size (PPS) sampling method. Finally, 20 households in each village were selected from the listing of households by systematic random sampling. In any selected village where 20 households were not available, the remaining households were covered from a nearby village. This was the case for three villages; therefore, three more villages were added. In addition, a few extra households were sampled during the course of data collection due to precautions taken by the investigators to avoid under sampling. Therefore, at the end of the study, a total of 1010 households were included in the survey, all of which were included in the analysis (also shown in Fig. 1).

All surveys were conducted in Hindi, the most common language locally, by trained interviewers with graduate-level educational qualification. Structured interview schedule was designed to interview the head of the each household to collect data on educational status and occupation of the family members, knowledge related to malaria aetiology, mode of transmission, mosquito breeding places, prevention and treatment-seeking practices of the heads of the households using recall method for 2 weeks reference period of febrile illness. Availability of health infrastructure nearby the studied villages and distance from village was observed and recorded by the interviewers from various sources. As the UMPs were not legally-authorized health care providers, they were difficult to identify and approach for the study. Therefore, household respondents were asked to help the investigators identify UMPs who were providing health care services in the community. Once a group of UMPs were identified, they were further used through snowballing technique to identify and recruit additional UMPs. Surveys with UMPs used a semi-structured, pre-tested interview schedule consisting variables to inquire their educational status, knowledge related to malaria aetiology, mode of transmission, common species of human plasmodia and their methods of diagnosis and treatment practices related to malaria. Average number of patients treated and the cost of treatment per patient was estimated based on responses reported by UMPs during interview using recall method for 1 week preceding the survey as reference period.

### Data entry and analysis

Survey interview schedules were checked for completeness; illogical or inconsistent responses were edited before leaving the field site. Random spot checks and back-checks during interviews were conducted to ensure data quality control. Data were double key-entered in CSPro 7.1.3 (US Census Bureau) with built-in data entry application and edit checks for quality control. Data were then exported to R v3.5.0 for Windows (R foundation for statistical computing) for statistical analysis. Numerically coded categorical variables were cross-tabulated in frequency and percentage distribution and continuous variables were summarized in mean and standard deviation (SD).

## Results

### Demographic and Socio-economic characteristics of the households

A total of 1010 households were surveyed, of which 85% belonged to different tribal groups. ‘Baiga’ tribe in Balaghat and Shahdol, ‘Bharia’ tribe in Chhindwara, ‘Saharia’ in Sheopur, and ‘Gond’ tribe, another dominant tribal group in all four districts except Sheopur, were represented in the study. The average family size was 5.6 per household, which was slightly higher than the average state-wide family size (4.7 persons per household) [13]. The sex ratio of the household members was 982 females per 1000 males, which was also higher than sex ratio of the state (931), as reported in census 2011 [13]. The literacy rate of household members above 6 years of age was 69%, below the state-reported rate of 71%, reported in census 2011 [13]. Agriculture (54%) and casual labour (43%) were the main occupations of residents. Only a small proportion of the population was engaged in salaried jobs (3%) or small businesses (1%) (Table 1).

**Table 1:** Education and occupation of study population and UMPs

Variables	Households (N = 1010)	UMPs (N = 140)
	n (%)	n (%)
Educational status (above 6 years of age)	N = 4552	
Illiterate	1412 (31.0)	0
Primary	1277 (28.0)	0
Middle	915 (20.1)	0
High school	601 (13.2)	21 (15.0)
Higher secondary	235 (5.2)	53 (37.9)
Graduate	112 (2.5)	34 (24.3)
Post graduate	0	9 (6.4)
Diploma/certificate	0	9 (6.4)
Other unspecific	0	14 (10.0)
Major occupation (above 14 years of age)	N = 3709	
Labourer	1577 (42.5)	na
Agriculture	1989 (53.6)	na
Salaried job	105 (2.8)	na
Business	38 (1.0)	na

na: not applicable

### Common ailments, knowledge, prevention and treatment-seeking practices of the households for malaria

Fever, cold, cough, and diarrhoea were the most common health problems reported by heads of households in the study area. Misconceptions related to the mode of transmission of malaria were very common. Less than half of the heads of households knew that mosquito bites were responsible for the spread of malaria in the community. Furthermore, regarding breeding places of mosquitoes, about two-thirds of heads of households responded that mosquitoes breed in mud, swamps, cow dung, forests, bushes, and shrubs. A higher proportion were aware that fever with chills and rigor, headaches, body aches, and vomiting are the main symptoms related to malaria. About 75% of the heads of households considered malaria a serious disease and believed it could be fatal if not treated promptly (72%) (Table 2).

**Table 2:** Knowledge of malaria aetiology among community and UMPs

Variables	Households (N = 1010)	UMPs (N = 140)
	n (%)	n (%)
Main symptoms of malaria		
Fever with chills & rigor	932 (92.3)	130 (92.9)
Headache, bodyache	895 (88.6)	124 (88.6)
Vomiting, nausea	830 (82.2)	131 (93.6)
Unconsciousness	0	5 (3.6)
Diarrhoea	268 (26.5)	0
Cold & cough, throat sore, runny nose	535 (53.0)	0
Jaundice	86 (8.5)	4 (2.9)
Mode of transmission of malaria		
Mosquito bite causes malaria	409 (40.5)	110 (78.6)
Other fly, bedbug, contaminated food/water etc.	601 (59.5)	30 (21.4)
Mosquito breeding place		
Mud, swamp, cow dung	455 (45.0)	78 (55.7)
Water	390 (38.6)	62 (44.3)
Forest, shrubs	165 (16.3)	0
Preventive measures		
Sleeping under bednet	571 (56.5)	47 (33.7)
Elimination of mosquito breeding places	86 (19.6)	93 (66.3)
Smoke burning leaves and cow dung	661 (65.4)	27 (19.3)
Mosquito repellent like coil cake	13 (1.3)	109 (77.9)
Indoor residual spray with insecticide	289 (28.6)	11 (7.9)
Common ailments in the area		
Fever	875 (86.6)	136 (97.1)
Cold & cough	635 (62.9)	35 (25.0)
Diarrhoea	578 (57.2)	57 (40.7)
Skin disease	166 (16.4)	11 (7.9)
Tuberculosis	104 (10.3)	8 (5.7)
Eye disease	29 (2.9)	0
Jaundice	226 (22.4)	2 (1.4)
Other (typhoid, viral fever)	154 (15.2)	15 (10.7)
Malaria a serious health problem	755 (74.8)	128 (91.4)
Malaria is fatal	724 (71.7)	131 (93.6)
Species of human malaria parasite		
*P. falciparum*	–	115 (82.1)
*P. vivax*	–	15 (10.7)
*P. malariae*	–	0
*P. ovale*	–	0
*P. knowlesi*	–	0
*P. falciparum* and *P. vivax*	–	10 (7.2)
Wrong answer i.e. filaria, dengue etc.	–	9 (6.4)
Most common species in the area		
*P. falciparum* only	–	124 (88.6)
*P. vivax* only	–	3 (2.1)
*P. falciparum* and *P. vivax* both	–	4 (2.9)
Wrong answer i.e. filaria, dengue etc.	–	9 (6.4)
Methods for malaria diagnosis		
Blood test by RDT only	–	2 (1.4)
Blood test by microscopy only	–	2 (1.4)
Blood test by RDT and microscopy both	–	136 (97.1)

–: not collected from household at community level

Most heads of households (> 90%) believed in the efficacy of indoor residual spraying of insecticide for malaria control. However, it was a normal practice in the area to paint the dwellings twice a year, mainly after the rainy season and in summer, which likely reduced the residual effect of the insecticide sprayed. Ownership of bed nets was highest in households residing in Balaghat (97%), followed by Betul (69%), Shahdol (46%), Chhindwara (45%) and Sheopur (3%). This difference may be due to the distribution of bed nets under the government’s malaria control programme, which was further confirmed by household heads and programme officials. Unaffordability due to the poor economic condition of most households was the major reason for not owning a bed net. For protection from mosquito bites, most households produced smoke by burning leaves and cow dung (65%), used cloths to cover most of the body (16%), and rubbed mustard oil on the body (23%). A smaller proportion (36%) of households practiced modern preventive measures like regular use of bed nets, mosquito repellents, coils, and cakes (Table 3).

**Table 3:** Malaria prevention and treatment seeking practices among community

Variables	n/d (%)
Prevention practices from mosquito bite	
House was sprayed with insecticide (IRS)	289/1010 (28.6)
IRS including kitchen	227/289 (78.5)
Household like IRS	983/1010 (97.3)
Household feels that IRS is effective	954/1010 (94.5)
House was whitewash (Mean ± SD) per year	1.9 ± 0.7
Household heard about insecticide treated bednets	435/1010 (43.1)
Household owned bednet	571/1010 (56.5)
No. of bednet (Mean ± SD) per household	1.6 ± 0.9
Reasons for not owing/using bednet (N = 439)	
Economic	321/439 (73.1)
Mosquito not bite	86/439 (19.6)
Feel uncomfortable	32/439 (7.3)
Owned insecticide treated bednet (N = 571)	438/571 (76.7)
Bednet provided by the government agency (N = 571)	438/571 (76.7)
Using any preventive measures to protect from mosquito bite	873/1010 (86.4)
Sleeping under bednet regularly	360/1010 (35.6)
Using mosquito repellent coil, cake, cream	13/1010 (1.3)
Smoke formation by burning leaves, cow dung	661/1010 (65.4)
Roping body oil	236/1010 (23.4)
Cover body	165/1010 (16.3)
Other	0
Any one suffered from suspected malaria in 2 weeks preceding the survey^a^	390/1010 (38.6)
Initial source of treatment sought (N = 390)	
Faith/traditional healer	57/390 (14.6)
Unqualified health providers	193/390 (49.5)
ASHA/ANM/health worker	57/390 (14.6)
PHC/CHC govt hospital	58/390 (14.9)
Private hospital	25/390 (6.4)
Malaria diagnosis/treatment is available within the village	599/1010 (59.3)
Treatment was freely provided	452/599 (75.5)
Availed free malaria treatment within the village	422/599 (70.4)
Satisfied with provided free malaria treatment	402/599 (67.1)

n/d: Numerator/denominator

^a^Refers to reported malaria

UMPs were the most preferred health providers (49%) in the community, and were residents’ first choice for seeking treatment of ailments. About 15% of the households sought treatment from faith/herbal healers, 15% went to community health workers like ASHAs, Auxiliary Nurse Midwives (ANMs), 15% visited government hospitals like primary health centres (PHCs) or community health centres (CHCs), and 6% went to private hospitals as their first choice for treatment during an illness. However, 60–70% of the household heads believed that diagnosis and treatment of febrile illness was freely available within the village and was provided by the ASHAs, which contrasts sharply with their practices (Table 3).

### Health infrastructure availability

Integrated child development scheme (ICDS) services by the Government of India through ICDS centres and its workers within the villages were available to more than 90% of the villages. ASHAs were providing primary health services in over 80% of the villages. However, peripheral community health providers like ANMs, health sub-centres, and village level health committees were less available in the villages (11–18%). The average distance of any government health facility such as PHCs and CHCs from the villages was 13.2 (SD: 1.9) and 28.6 (SD: 10.1) km, respectively. The mean distance of public bus stops from villages was recorded to be 6.4 (SD: 3.6) km (Table 4). Public transportation was very poor in the study areas.

**Table 4:** Health infrastructure availability in study villages

Variables	(N = 53)
	n (%)
Health Infrastructure available in the villages	
Aagnawadi (ICDS) centre	52 (98.1)
Aaganwadi (ICDS) assistant	48 (90.6)
Aaganwadi (ICDS) worker	48 (90.6)
Aaganwadi (ICDS) is functioning regularly	48 (90.6)
ASHA worker is residing in the village	45 (84.9)
Health sub-centre available in the village	10 (18.9)
Nurse is residing in the village	6 (11.3)
Health committee is functioning in the village	10 (18.9)
Distance of Health facilities from the village	
PHCs (mean ± SD) km	13.2 ± 1.9
CHCs (mean ± SD) km	28.6 ± 10.1
Distance of bus stop (mean ± SD) km	6.4 ± 3.6
Vehicle available in emergency in the villages	35 (66.0)

### Knowledge, perception, and practices of UMPs

A total of 168 UMPs were identified of which 28 were not willing to participate in the study, rest 140 UMPs were interviewed. All the UMPs were male with an average age of 33.3 (SD: 8.1) years. About 50% of the participants had completed up to secondary level of school education, 24% were graduates (with non-medical degree), 6% were post-graduates with non-medical degrees, and 6% had acquired a short term certificate or diploma in Medical Laboratory Technology, Certificate of Training of “Jan Swasthya Rakshak” (Village Health Guide). The remaining 10% had other training certificates from unrecognized agencies or organizations (Table 1).

The UMPs had 2–15 years of experience (average of 8.3; SD: 3.1 years) providing health care services and were treating patients mostly for common illness such as malaria, typhoid, cold and cough, diarrhoea, and skin diseases. Most (79%) of the UMPs knew about the aetiology (cause and clinical symptoms) of malaria. However, the remaining 21% of the UMPs had misconceptions about the mode of transmission of the disease and reported that house flies, bedbugs, and consuming contaminated water or food also caused malaria infection. More than 50% of the UMPs were not aware of the proper breeding places of mosquitoes and lacked knowledge regarding modern preventive measures for malaria (Table 2). Knowledge about human plasmodia was also inadequate; 82% of the UMPs knew only *P. falciparum* species, 11% knew only *P. vivax,* 7% knew both *P. falciparum* and *P. vivax*. Around 6% reported filaria and dengue as names of species of *Plasmodium* (Table 2).

The average charge for the diagnosis and treatment of any disease was 4.27 USD per patient, which varied with type of the disease and patient condition. In 1 week before the survey, an average of 47.3 (SD: 34.9) patients were treated by the UMPs, of which 27.4 (SD: 24.7) patients had febrile illness and were suspected to be infected with malaria. Overall, 38% of UMPs admitted that they usually provide anti-malarial treatment to the suspected malaria cases without confirmed diagnosis; the remaining 62% mentioned that they provide treatment only after blood test using malaria rapid diagnostic test kit (RDT). Some of most recently used RDTs were physically verified by the interviewers during the survey. The malaria RDTs were procured from the local market. Injectable modes of treatment were most favoured (67%) as per the choice exercised by the patients. Only 9% of the UMPs had heard about the national policy for diagnosis and treatment of malaria, though none had actually read the guidelines. Four percent of the UMPs mentioned that they had also treated serious and complicated malaria cases, which is not advised by the guidelines (such cases should refer to nearest health facilities).

Regarding the anti-malarial treatment practices for different parasite species, none of the UMPs adhered to the national drug policy; frequently they practiced monotherapy of artemisinin. It was also observed that *P. falciparum* cases were also treated with chloroquine, despite resistance in most part of the country. Small proportions of cases were also treated with quinine, which is not advisable in field conditions and requires strict medical supervision as per guidelines. Only 18% of *P. falciparum* and 5% *P. vivax* cases were provided primaquine, creating opportunities for further transmission of malaria in the community. The use of inappropriate regimes of anti-malarials with inadequate dosages for treatment of suspected and/or confirmed malaria cases was commonly observed and was noticed as a serious concern (Table 5).

**Table 5:** Treatment practices of UMPs particularly for malaria

Variables	N = 140
	n (%)
Total length (mean ± SD) of medical practice (year)	8.3 ± 3.1
Attended any training programme	125 (89.3)
Training provided by govt. agency	40 (28.6)
Training period (months) (mean ± SD)	7.3 ± 3.5
Training on malaria diagnosis and treatment	6 (4.3)
Major common diseases in area	
Malaria	136 (97.1)
Diarrhoea	57 (40.7)
Skin disease	11 (7.9)
Cough cold	35 (25.0)
Typhoid	6 (4.3)
Tuberculosis	8 (5.7)
Viral Fever	9 (6.4)
Jaundice	2 (1.4)
Providing services on following health issues	
Maternal health care	72 (51.4)
Child health care	106 (75.7)
Family planning	124 (88.6)
Vaccination	111 (79.3)
Common diseases (diarrhoea, skin diseases, typhoid, piles, asthma)	140 (100.0)
Malaria	138 (98.6)
Tuberculosis	5 (3.6)
No. of patients treated in last week (mean ± SD)	47.3 ± 34.9
No. of fever cases treated in last week (mean ± SD)	27.4 ± 24.7
No. of malaria patient treated in last week (mean ± SD)	17.6 ± 5.8
Usually treat suspected malaria cases based on clinical symptoms	53 (37.9)
Treat suspected malaria cases only after blood test using RDTs	87 (62.1)
Having malaria RDT in stock (physically verified)	127 (90.7)
Source of RDT procured	
Market	137 (97.9)
Government Hospital/ASHAs	2 (1.4)
Source of antimalarial procured	
Market	138 (98.6)
Government hospital/ASHAs	2 (1.4)
Mode of treatment preferred	
Injectable	94 (67.1)
Oral (tablets/syrup)	15 (10.7)
Both	31 (22.1)
Cause of preference of injectable	
Patient psychology (client’s preference)	103 (73.6)
Fast recovery (provider’s preference)	37 (26.4)
Treated complicated/serious malaria patient	6 (4.3)
Heard about national diagnosis and treatment guidelines of malaria	12 (8.6)
Treatment practices of malaria patients	
E-Mal (artesunate injection)	
*P. falciparum*	95 (67.9)
*P. vivax*	17 (12.1)
Quinine tablets	
*P. falciparum*	2 (1.4)
*P. vivax*	3 (2.1)
Quinine injection	
*P. falciparum*	2 (1.4)
*P. vivax*	2 (1.4)
Chloroquine tablets	
*P. falciparum*	18 (12.9)
*P. vivax*	31 (22.1)
Chloroquine injection	
*P. falciparum*	11 (7.9)
*P. vivax*	33 (23.6)
Primaquine tablets	
*P. falciparum*	25 (17.9)
*P. vivax*	7 (5.0)

## Discussion

Malaria incidence was most pronounced in tribal dominated areas of the country [6]. These tribal dominated areas are hilly, forested, and inaccessible in much of the rainy season, which is the main transmission season for malaria [15]. Tribal communities preferred informal health providers, such as faith/traditional healers and unqualified or unlicensed medical practitioners. This preference is less by choice than by necessity due to low literacy levels, poor economic conditions, and inadequate health infrastructure which act to limit awareness of disease transmission and prevention, diagnosis, and treatment [6, 7, 11]. UMPs are the first source of treatment providers for more than half the population in rural tribal areas [7, 11, 12]. Although UMPs lack medical degrees, they have self-acquired knowledge and experience administering allopathic medicines.

Despite their knowledge and experience, the survey results found many examples of inappropriate treatment practices. UMPs frequently treated *P. falciparum* patients with monotherapy of artemisinin, often with incomplete doses which can create drug pressure and lead to drug resistance [16]. It has also been reported in other studies that artemisinin mono-therapy has been prescribed by qualified allopathic and non-allopathic physicians for treatment of uncomplicated malaria in other areas [17]. UMPs also treated *P. vivax* patients with artemisinin, while many *P. falciparum* patients were treated with chloroquine, which is against the national treatment guidelines for malaria [18]. In addition, national guidelines state that quinine is only to be given to serious and complicated confirmed malaria patients under strict medical supervision [18].

This study confirmed that the poor health infrastructure in remote rural and inaccessible tribal dominant areas of the state compels community members to seek treatment from unlicensed private health providers, mainly because they cannot afford wage loss due to sickness [19]. Alternative health services are only available at locations too far away to be accessible [20]. Thus residents in these areas represent a vulnerable population unable to access malaria diagnosis and treatment services that adhere to national guidelines. While they do not receive the quality of services obtained by residents in other parts of India, their ability to access UMPs provides them with providers who are described as hospitable and able to provide hassle-free, relatively low-cost medicines and services. These findings conform with those of a study conducted by Das and Mohpal in Madhya Pradesh, which revealed that most private providers in rural areas lack formal medical training, but they had spent more time with the patients and thus win over their trust [11].

While few of the UMPs acknowledged that they treated serious and complicated malaria cases instead of referring them to the nearest health facility, it is important to recognize that such treatment represents an important contribution given the scarcity of government health infrastructure in remote rural areas. This lack of trained human resources is an important factor for poor health delivery in rural areas. The educational background and skills of UMPs related to diagnosis and treatment might be stronger than those of ASHAs, who are primary health care providers in rural communities. Therefore, it is recommended that UMPs be trained as per the national guidelines for diagnosis and treatment of common illnesses, including malaria, to enhance their ability to play a significant role to achieving India’s malaria elimination goals.

In 2009, a 6 months’ training programme was organized in the state of Andhra Pradesh to develop treatment skills of common ailments and awareness among UMPs [21]. Similarly, in the state of Chhattisgarh, a three-year medical diploma course was initiated in 2001 to train health practitioners for rural areas. However, these programmes were aborted, mainly due to resistance from the Medical Council of India (MCI), the professional body regulating medical education in India. The MCI raised the issue that approval to practice medicine by UMPs may promote the irrational use of allopathic drugs and bring a medical catastrophe. In July 2019, the Government of India introduced National Medical Commission Bill, 2019, in assembly which may grant a limited license to certain mid-level practitioners (community health providers) connected with the modern medical profession to practice allopathic medicine. These mid-level practitioners may prescribe specified medicines in primary and preventive healthcare. In other situations, these practitioners may only prescribe medicines under the supervision of a registered medical practitioner [22].

## Conclusions

Developing a well-designed training protocol for UMPs, particularly on anti-malarial treatment, may be useful in areas of India, along with limiting their role to be similar to that of health volunteers such as ASHAs and other programme-driven village-level health care providers, rather than giving them the status of a doctor. In addition to training and equipping UMPs to provide basic health services, including anti-malarial services, close monitoring of their capabilities should be enforced to ensure their credibility. Such activities, designed to incorporate trained UMPs into the existing programme for achieving India’s malaria elimination goals in hard-to-reach tribal areas, can only bring positive results.

## Limitations

All efforts were made to capture valid responses from the UMPs who participated in the study. Responses were collected only from UMPs who volunteered for the survey, and this might have resulted into certain amount of biasness. However, such bias was not so extensive and thus this possibility is not overly concerning.

## Data Availability

The raw data collected and used in the present analysis will be available from the corresponding author on reasonable request.

## Abbreviations

ASHAs: Accredited Social Health Activists
UMPs: unlicensed medical practitioners
WHO: World Health Organization
ACT: artemisinin based combination therapy
API: annual parasite incidence
PPS: probability proportional to size
SD: standard deviation
ANMs: auxiliary nurse midwives
PHCs: primary health centres
CHCs: community health centres
ICDS: integrated child development scheme
RDT: rapid diagnostic test
MCI: Medical Council of India
